# The Role of Pericardial Fat in Cardiometabolic Disease: Emerging Evidence and Therapeutic Potential

**DOI:** 10.1007/s11886-025-02305-9

**Published:** 2025-10-10

**Authors:** Zaid Shahrori, Michel Chedid El Helou, Sherin Sallam, Gianluca Iacobellis, Ian J. Neeland

**Affiliations:** 1 Department of Medicine, Case Western Reserve University School of Medicine, Cleveland, OH USA; 2 Department of Medicine, University Hospitals Cleveland Medical Center, Cleveland, OH USA; 3 Harrington Heart and Vascular Institute, University Hospitals, Cleveland, OH USA; 4 Division of Endocrinology, Diabetes and Metabolism, University of Miami, Miami, USA

**Keywords:** Visceral adipose tissue, Ectopic fat, Artificial intelligence, Deep learning, Cardiovascular risk, Medical image segmentation

## Abstract

**Purpose of Review:**

This review explores the unique anatomy and pathophysiology of pericardial fat (including both epicardial adipose tissue [EAT] and paracardial fat), its clinical significance, and its potential role as a therapeutic target. It addresses key questions regarding the contribution of EAT to cardiometabolic conditions such as coronary artery disease, heart failure, atrial fibrillation, and ventricular arrhythmias, and explores interventions that reduce EAT to possibly improve cardiovascular outcomes.

**Recent Findings:**

Recent studies have established EAT as a metabolically active, pro-inflammatory fat depot directly affecting the myocardium and coronary arteries. Imaging and metabolomic studies have advanced the assessment of EAT burden. Clinical evidence supports lifestyle modification, pharmacologic therapies including GLP-1 RA and SGLT2i, and bariatric surgery to effectively reduce EAT volume. Emerging data link EAT reduction with improved cardiac function and arrhythmia risk, although causality remains unclear.

**Summary:**

EAT is a modifiable cardiometabolic risk factor associated with adverse outcomes in coronary artery disease, heart failure, atrial fibrillation, and ventricular arrhythmias. Targeting EAT through cardiometabolic risk reduction strategies may improve prognosis. Future research should focus on determining whether reducing EAT directly improves clinical outcomes.

## Introduction: Anatomy and Physiology of Pericardial Fat

The anatomical classification of “pericardial fat” actually includes three distinct fat depots which are embryologically, anatomically, physiologically, and clinically distinct (Table 1). Epicardial adipose tissue (EAT) is located on the surface of the myocardium within the pericardial sac under the visceral pericardium [1]. Pericardial fat is technically the depot located between the visceral and parietal pericardium, although the term is often used with less precision and interchangeably in the literature referring to the combination of adipose tissue layers around the heart [1]. Paracardial fat (sometimes called extra-pericardial fat) lies outside the fibrous parietal pericardium, within the mediastinum [1].

**Table 1:** Imaging modalities for assessment of pericardial fat: advantages and limitations

Modality	What is Measured	Advantages	Limitations	Key References
Echocardiography	EAT thickness (mm) on RV wall	Widely available; no radiation; inexpensive; quick. Can roughly track changes over time.	Limited to 2D thickness (cannot measure total volume); less accurate in obesity (image quality); operator dependent.	[16–18]
Computed Tomography (CT)	EAT and/or paracardial fat volume (cm³), fat area or thickness. Often obtained from non-contrast cardiac CT or CT angiography	High spatial resolution; 3D quantification of volume and distribution; highly reproducible; can simultaneously assess coronary calcium or CAD.	Involves ionizing radiation; requires image segmentation (labor-intensive or need automated software); not ideal for repeated scans.	[20–22]
Magnetic Resonance (MRI)	EAT volume (cm³); fat thickness; can characterize fat vs. fibrous tissue	No radiation; excellent tissue characterization and volume accuracy (reference standard); reproducible with low observer variability.	High cost and less accessible; longer scan times; specialized sequences needed; not typically used in routine practice for fat measurement.	[2,23]

*2D* 2-dimensional, *3D* 3-dimensional, *CAD* coronary artery disease, *CT* computed tomography, *EAT* epicardial adipose tissue, *MRI* magnetic resonance imaging, *RV* right ventricular

Anatomically, EAT is distributed over the heart surface, most commonly found near the atrioventricular and interventricular grooves and along the course of the coronary artery [2]. Smaller fat deposits can also be found adherent to the atrial free wall [2]. In individuals with normal weight, EAT constitutes roughly 15–20% of total cardiac weight, but this can increase substantially in obesity [3]. Epicardial fat is a true visceral fat depot and derives from splanchnic mesoderm (brown-fat lineage) and is composed of adipocytes, nerves, inflammatory cells, and stromal tissue. There is no separating structure between EAT and the myocardium or adventitia of coronary vessels, so epicardial fat is in direct contact with the heart’s surface and it shares the heart’s microcirculation [3]. A direct connection between EAT and the coronary wall via vaso vasora has also been studied [4].

By contrast, pericardial fat originates from a different embryologic precursor (parietal mesoderm) and is supplied by non-coronary arteries (such as branches of the internal thoracic artery) [2]. Paracardial fat derives from the thoracic mesenchyme and is a part of thoracic extra-pericardial fat, more akin to other body fat depots, and does not directly contact the myocardium [2].Under physiological conditions, paracardial fat typically accumulates in regions like the anterior cardiophrenic angles (pericardial fat pads) and can account for up to 20–50% of total cardiac fat mass in normal weight individuals. Because of these anatomical differences, EAT, pericardial fat, and paracardial fat have distinct functional and pathophysiologic roles despite their proximity [2]. This review will focus primarily on EAT give its direct continuity with the myocardium and coronary bed.

## Pathophysiology of Epicardial Fat in Cardiometabolic Disease

EAT is a metabolically active organ that directly interfaces with the myocardium and cardiac circulation, influencing the heart through paracrine and mechanical effects. EAT can serve as an energy depot, aiding thermogenesis, and producing various peptides [5]. However, it can also contribute to adverse mechanisms in cardiometabolic disease, several of which have been described in the literature and will be discussed below.

### Inflammatory Cytokine Secretion

EAT contains inflammatory cells, such as macrophages, and is capable of secreting pro-inflammatory cytokines and adipokines that diffuse into cardiac tissues such as interleukin (IL)−6, tumor necrosis factor (TNF)-α, monocyte chemoattractant protein-1 (MCP-1) [5]. EAT also expresses adipokines like resistin and leptin at high levels. In acute coronary syndromes, resistin expression in EAT is elevated and may promote endothelial dysfunction and plaque instability [6]. Given the close proximity to the underlying myocardium and coronary arteries, EAT-derived factors can diffuse into these areas, potentially accelerating atherosclerosis, endothelial dysfunction, and fibrosis in a paracrine fashion.

### Oxidative Stress and Lipotoxicity

EAT undergoes lipolysis, releasing free fatty acids, and when it there is overactive lipolysis (such as in obesity) excess free fatty acids can result in lipotoxicity from increased reactive oxygen species. Fatty infiltration of the myocardium has been observed in individuals with extremely high EAT. In obesity, epicardial fat cells may enlarge and become dysfunctional, reducing their ability to buffer fatty acids. This can increase fatty acid spillover into the heart muscle and circulation, leading to myocardial steatosis and impaired contraction. Excess EAT can surround the heart, limit diastolic filling, and elevate ventricular pressures, contributing to features of heart failure with preserved ejection fraction (HFpEF) [7].

### Fibrosis and Autonomic Effects

EAT also contributes to myocardial fibrosis and autonomic dysfunction by releasing pro-fibrotic mediators like TGF-β, CTGF, Activin A, and Angptl2, all linked to atrial fibrosis and arrhythmogenic substrates [8]. Studies demonstrate that expansion of EAT, particularly around the atria, is linked to atrial myocardial fibrosis and conduction slowing, creating a substrate for atrial fibrillation (AF) [9]. Furthermore, EAT harbors ganglionated plexi, part of the cardiac autonomic nervous system, and may influence autonomic tone [8]. Through this interaction, increased EAT contributes to heightened sympathetic activity, vagal imbalance, and heterogeneous conduction within atrial tissue. Fibro-fatty infiltration from EAT further slows conduction velocity in adjacent myocardium, creating reentrant circuits and promoting arrhythmogenesis [8].

### Pathophysiology of Pericardial and Paracardial Fat

Although pericardial and paracardial fat are less studied than EAT, these depots are metabolically active, and their dysfunction is associated with impaired mitochondrial biogenesis and increased pro-inflammatory signaling, The pathophysiology of pericardial and paracardial fat involves both local and systemic mechanisms. Pericardial and paracardial fat expand with aging, obesity, and hormonal changes (notably menopause), and are characterized by increased inflammatory cytokine secretion and altered adipokine profiles. These fat depots undergo remodeling with aging and high-fat diets, regulated by alcohol dehydrogenase 1 (ADH1) through retinaldehyde-mediated PGC-1α activation, promoting mitochondrial biogenesis and metabolic flexibility. ADH1 deficiency leads to paracardial fat expansion and cardiometabolic impairment, reversible by retinaldehyde supplementation or surgical fat removal [10].In addition, paracardial fat was shown to be strongly associated with coronary artery calcification in postmenopausal women compared to premenopausal women, independent of overall adiposity. This association is modified by estradiol levels, suggesting a menopause-specific role for paracardial fat in coronary risk [11].

### Metabolomics of Pericardial Fat

Several studies have investigated the associations between circulating metabolites and pericardial fat. In a study of healthy obese women, Scherer et al. found that higher epicardial fat volumes correlated with distinct plasma lipid profiles, including elevated diacylglycerols, triglycerides, and specific phospholipids and sphingolipids [12]. These findings suggest that lipidomic patterns in blood may reflect epicardial adiposity independent of other metabolic disease traits. Another study using data from the MESA and Rotterdam studies, further identified serum metabolites that were consistently associated with EAT volume, even after adjusting for body mass index and other clinical factors [13]. The metabolites identified were branched-chain amino acids, 1,5-anhydrosorbitol, and N-acetylated glycoproteins. Earlier work showed similar metabolomic signatures linked to visceral adipose tissue in the MESA and NEO studies [14], suggesting some overlap in the metabolic profiles of visceral and epicardial fat, but also pointing to specific markers for epicardial fat. These studies provide insights into the metabolic mechanisms and pathways relating pericardial fat with cardiometabolic health.

## Assessment of Pericardial Fat

Accurate measurement of epicardial, pericardial, and paracardial fat can be achieved with several imaging modalities [15]. The most often used techniques are echocardiography, computed tomography (CT), and magnetic resonance imaging (MRI), each with distinct advantages and limitations (Table 1). There is no standardized clinical protocol for routine pericardial fat measurement, but these tools are frequently used in research settings.

### Echocardiography

Transthoracic echocardiography visualizes epicardial fat as an echo-lucent space on the surface of the myocardium, which is usually best seen adjacent to the right ventricular free wall. Echocardiography is often used to measure epicardial fat thickness (in millimeters) at the end-systole on the right ventricular free wall [16]. This provides a simple surrogate for EAT volume. Echo-EAT thickness measurements demonstrate high reliability, with inter-observer ICC of 0.97 and intra-observer ICC of 0.99 when using maximal thickness at the Rindfleisch fold, an anatomical area located at the junction of the right atrium and right ventricle where epicardial fat is most prominent [17]. Other studies have reported moderate to good reliability, such as intra-observer ICC of 0.625 and inter-observer ICC of 0.605 using standard methods, while Iacobellis et al. found strong inter-observer ICC of 0.90 and intra-observer ICC of 0.98 [16, 18].

The major advantages of echocardiography are its wide availability, low cost, and absence of radiation. However, only fat thickness in a limited view and no measures of distribution can be observed. The accuracy suffers in obese patients, in which these measurements are of particular importance. Thus, while convenient, echocardiographic EAT measurement lacks precision and is best for semi-quantitative assessment or longitudinal tracking in an individual [19].

### Computed Tomography (CT)

*Cardiac* or chest CT provides high-resolution 3D visualization of adipose tissue around the heart. Typically, volumetric measurement of EAT is done by segmenting the heart’s pericardial contour on each slice and summing the fat voxels inside that contour. Epicardial pericardial fat are difficult to separate on CT imaging given their close proximity and thin fascial plane separating them. However, epicardial/pericardial fat can be differentiated from paracardial fat with CT by delineating the pericardial boundary. However, how this boundary is defined is an evolving field, and advances in imaging software and machine learning techniques have largely shifted measurements from manual to an automated process [20]. In both the Multi-Ethnic Study of Atherosclerosis (MESA) and Rotterdam Study, investigators found it difficult to separately quantify the epicardial and pericardial fat due to lack of consistently clear pericardial definition using the available imaging, especially in lean individuals. To address this issue, epicardial fat was specifically measured in a random sample of 159 MESA participants, and the Spearman correlation coefficient between total pericardial and epicardial fat was 0.92 (*P* < 0.0001); therefore total pericardial fat volume measurement has been used in several studies of the MESA cohort [21]. The strengths of CT are its high spatial resolution and ability to quantify volume and fat distribution objectively. CT-measured EAT volume has excellent inter-observer reproducibility, additionally, a single cardiac CT scan can assess coronary calcification or coronary artery disease concurrently with fat measurement [22]. The main limitation is radiation exposure, which makes CT less suitable for serial monitoring. CT imaging also requires gating and good breath-hold cooperation.

### Magnetic Resonance Imaging (MRI)

Cardiovascular MRI is widely considered the gold-standard for cardiac volume assessments given its ability to distinguish tissue (through fat-water separation imaging) and excellent visualization of visceral and parietal pericardium [23]. Its limitations are cost, lower availability, and longer scan times. MRI may be less practical for routine fat measurement, and small amounts of fat can sometimes be missed if spatial resolution is not high or if appropriate sequences are not used [2]. Many research studies nevertheless use MRI to validate EAT measurements. In practice, CT is more commonly used than MRI for EAT quantification, given its greater availability.

## Clinical Implications and Associations with Cardiometabolic Outcomes

**Fig. 1:**
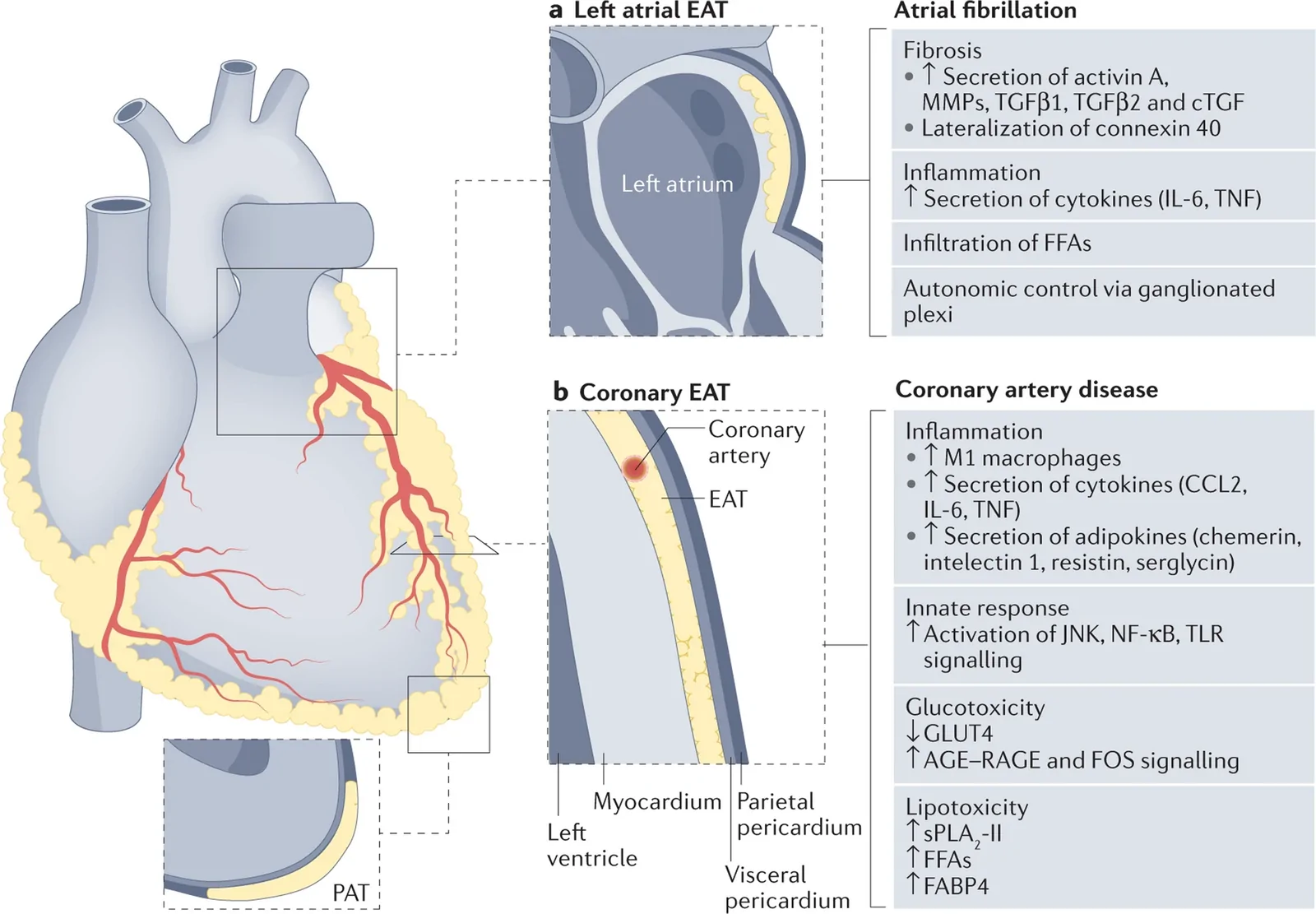
Role of regional EAT depots on coronary artery disease and atrial fibrillation. The epicardial adipose tissue (EAT) is distributed as localized depots lying between the myocardium and the visceral layer of the pericardium. EAT can infiltrate the left atrium (left atrial EAT) and surround the coronary arteries (coronary EAT). By contrast, pericardial adipose tissue (PAT) is located more externally, within the visceral and parietal layers of the pericardium. EAT contributes to the development and progression of coronary artery disease and atrial fibrillation through complex and multifactorial pathways. The regional distribution of EAT has an important role because each EAT depot is anatomically, genetically and functionally different. **(a)** Left atrial EAT has a high expression of genes encoding pro-arrhythmogenic factors. Left atrial EAT can contribute to atrial fibrillation through the local secretion of profibrotic factors (matrix metalloproteinases (MMPs), transforming growth factor-β1 (TGFβ1) and TGFβ2, connective tissue growth factor (cTGF) and activin A) and inflammatory factors (IL-6 and tumor necrosis factor (TNF)) as well as free fatty acid (FFA) infiltration and increased autonomic control via ganglionated plexi. **(b)** The coronary EAT has a high expression of genes encoding pro-inflammatory adipokines and factors regulating glucose and lipid metabolism. Coronary EAT can influence the development and progression of coronary artery disease through increased infiltration of pro-inflammatory M1 macrophages from EAT into the adjacent myocardium, the paracrine or vasocrine release of several pro-inflammatory cytokines (CCL2, IL-6 and TNF) and adipokines (chemerin, intelectin 1 (also known as omentin 1), resistin and serglycin), and the activation of innate immune response factors such as JUN N-terminal kinase (JNK), nuclear factor-κB (NF-κB) and Toll-like receptors (TLRs). Upregulation of signaling via advanced glycation end products (AGE) binding to their receptor RAGE in EAT can contribute to the oxidative stress and endothelial damage associated with coronary atherosclerosis in patients with diabetes mellitus. The excessive influx of FFAs from EAT into the coronary arteries is mediated by enzymes such as group II secretory phospholipase A_2_ (sPLA_2_-II) and adipocyte fatty acid-binding protein (also known as FABP4). GLUT4, glucose transporter type 4. (Reproduced from: Iacobellis G. Nat Rev Cardiol. 2022;19:593–606, with permission from Springer Nature) [4]

Based on the pathophysiologic mechanisms described above, pericardial fat in general, and EAT specifically, have been linked to coronary artery disease (CAD) and atrial fibrillation (Fig. 1), heart failure, and ventricular arrhythmias (Table 2).

**Table 2:** Associations between epicardial adipose tissue and major cardiac outcomes

Cardiac Condition	Key Findings	Association Strength	Key References
Coronary artery disease	Greater EAT volume is independently associated with coronary atherosclerosis, plaque burden, and increased risk of MACE.	HR 1.35–2.05 for MACE; OR 2.63 for MI; OR 2.99 for revascularization	[21,24]
Heart failure with preserved ejection fraction	Increased EAT is linked to diastolic dysfunction, myocardial stiffness, and an increase in HF hospitalization and mortality.	SMD 0.61 for higher EAT in HFpEF vs. controls; HR 1.28 for HF hospitalization/mortality	[26,27]
Atrial fibrillation	EAT expansion promotes atrial fibrosis and autonomic imbalance, increasing AF incidence and recurrence after ablation.	OR 4.04 for AF risk; RR 1.06–1.73 for recurrence	[24,28]
Ventricular arrhythmias (including ventricular tachycardia)	Greater EAT in AV grooves is linked to post-ablation VT recurrence and arrhythmia substrate formation.	HR 1.20 per 1 mm EAT increase (*P* = 0.004)	[30]

*AF* atrial fibrillation, *AV* atrioventricular,*EAT* epicardial adipose tissue,*HF *heart failure,*HFpEF* heart failure with preserved ejection fraction, *HR* hazard ratio,*MACE* major adverse cardiovascular events, *MI* myocardial infarction, *OR* odds ratio, *SMD* standardized mean difference,*VT* ventricular tachycardia

### Coronary Artery Disease

There is a growing body of evidence linking increased EAT with coronary atherosclerosis and ischemic heart disease risk. Mechanistically, EAT overlying the coronary arteries can promote local inflammation of the vessels. A meta-analysis pooling data from CT and echocardiography-based studies demonstrated that patients with obstructive CAD had significantly greater EAT volumes compared to controls, and higher risks of major adverse cardiac events (MACE; HR 1.42–2.05), myocardial infarction (OR 2.63), coronary revascularization (OR 2.99), and cardiac death (OR 2.53) [24]. Another single-center study found that higher EAT volume, measured by non-contrast CT, independently correlated with significant coronary artery disease; for every 10 cm³ EAT, the odds of CAD increased by 53%, especially above an EAT of 134.4 cm³. Similarly, Eisenberg et al. used deep learning on non-contrast cardiac CT from the EISNER trial and showed greater EAT volume was linked to increased MACE risk (HR 1.35), while higher EAT attenuation reduces this risk (HR 0.83); MACE risk increased further when EAT volume was ≥ 113 cm³ [25]. Collectively, these findings suggest that EAT burden, especially when measured volumetrically on CT, provides incremental risk stratification for CAD, independent of BMI and other factors.

### Heart Failure

EAT has been increasingly recognized as a crucial factor in the pathophysiology and prognosis of heart failure, especially heart failure with preserved ejection fraction (HFpEF). A meta-analysis including 23 studies found that EAT was significantly higher in patients with HFpEF compared with controls, with a standardized mean difference (SMD) of 0.61 (95% CI: 0.27–0.94). However, in heart failure with reduced ejection fraction (HFrEF), EAT was not significantly different from controls and was inconsistently associated with cardiac function parameters [26]. Another meta-analysis of 9 studies found that increased EAT was associated with a higher risk of heart failure hospitalization and all-cause mortality, with a hazard ratio (HR) of 1.28 per unit increase (95% CI: 1.12–1.45, *p* = 0.0002) [26]. The association was particularly significant in patients with HFpEF, while evidence was inconsistent in those with HFrEF [27].

### Atrial Fibrillation

Evidence for the effects of EAT on incidence and recurrence of atrial fibrillation (AF) has been accumulating. A recent meta-analysis of 10 studies involving 1,840 patients with AF found that increased EAT was linked to a higher risk of AF recurrence after catheter ablation (RR = 1.06, 95% CI 1.02–1.11, *P* = 0.005), and greater EAT thickness was also identified as a risk factor (RR = 1.73, 95% CI 1.04–2.87, *P* = 0.040) [28]. A meta-analysis of 29 studies (*n* = 19,709) found that higher EAT volume and thickness were independently linked to an increased risk of MACE, with adjusted hazard ratios of 1.74 per 10 cm³ increase in CT-measured volume and 1.20 per 1 mm increase in echocardiographic thickness. Increased EAT was also associated with greater odds of cardiac death (OR 2.53), myocardial infarction (OR 2.63), coronary revascularization (OR 2.99), and atrial fibrillation (adjusted OR 4.04) [24]. These studies suggest that EAT may contribute to AF pathogenesis through atrial fibrosis, autonomic dysfunction, and inflammation. Clinically, EAT quantification enhances AF risk stratification and predicts post-ablation recurrence, supporting interventions that reduce EAT burden.

### Ventricular Arrhythmias

Although data are more limited, increasing evidence suggests a role for EAT in ventricular arrhythmogenesis. In patients undergoing ventricular tachycardia (VT) ablation, increased EAT thickness in the atrioventricular (AV) grooves correlated with higher recurrence rates. Right AV groove EAT ≥ 15.5 mm predicted lower VT-free survival (*P* = 0.003), and each 1 mm increase raised VT recurrence risk by 20% (HR 1.20 per mm, 95% CI 1.06–1.39, *P* = 0.004) [29]. Excess EAT increases post-ablation recurrence, possibly due to fibrosis, fat infiltration, or electrophysiologic changes. Assessing EAT may help stratify risk in ablation patients.

However, it remains unclear whether EAT is a direct cause of these conditions or an indicator of underlying risk. While studies suggest a contributory role, randomized trials are needed to confirm if reducing EAT improves outcomes.

## Therapeutic Targets and Management of Pericardial Fat

Given the link between EAT and adverse cardiac outcomes, there is growing interest in therapies that reduce EAT to mitigate its potential effects on cardiometabolic risk. Key approaches include lifestyle modification, weight loss therapies, and pharmacologic agents [30]. While no medication has been specifically studied to target EAT nor is officially approved for EAT reduction, several therapies have shown the ability to decrease EAT in secondary analyses of randomized controlled trials.

### Lifestyle Interventions

Lifestyle modification is fundamental for patients with excess visceral and ectopic fat, including EAT, with weight loss through diet being a first-line approach. Caloric restriction and reduced energy diet leading to weight loss have been shown to significantly reduce EAT thickness. In a study of patients living with severe obesity, a 20% reduction in body weight led to a 32% decrease in EAT thickness [31]. Notably, the reduction in EAT was proportionally greater for EAT than for generalized adiposity, and the EAT reduction correlated with improvements in left ventricular mass and diastolic function [31]. These findings suggest EAT may be especially responsive to weight loss similar to other “visceral” fat depots. Furthermore, a meta-analysis of multiple weight-loss therapies including lifestyle modifications, pharmaceuticals, and surgical options showed a decrease in EAT across all modalities [32].

Regular physical activity complements diet in managing EAT. Exercise can reduce EAT volume even in the absence of significant weight change [31]. A meta-analysis of exercise interventions found that exercise significantly decreased EAT (SMD ~ − 0.57) and waist circumference despite minimal change in body weight or BMI [33]. In other words, exercise appears to selectively mobilize visceral fat stores like EAT and abdominal fat, likely by improving muscle insulin sensitivity and increasing fat oxidation. Both aerobic exercise and resistance training have been associated with EAT reduction, with some evidence that higher-intensity aerobic exercise may confer larger reductions in EAT volume. Clinically, encouraging at least 150 min of moderate or 75 min of vigorous exercise per week (per guideline recommendations) is prudent [34]. Such lifestyle interventions not only reduce EAT but also improve blood pressure, glycemic control, and inflammatory markers, benefiting cardiovascular health.

### Pharmacologic Interventions

Several drug therapies target EAT indirectly by modulating metabolic drivers of adiposity or by exerting effects on adipose tissue distribution. Recent clinical trials and meta-analyses highlight the impact of both traditional cardiovascular drugs and newer metabolic agents on EAT. Multiple studies have examined the role of lipid lowering therapies on EAT volume and density. A study by Raggi et al. demonstrated that statins induced a decrease in EAT density while not affecting subcutaneous fat density suggesting the effects might be mediated by a reduction in inflammation, cellularity, or vascularity. A small observational study showed a 20.4% decrease in EAT thickness after 6 months of treatment with PCSK-9 Inhibitors [35]. Another study showed that PCSK9 concentration was linked to EAT volume in patients with type 1 diabetes [36].

Glucagon-like peptide-1 receptor agonists (GLP-1 RAs) and sodium-glucose cotransporter 2 inhibitors (SGLT2i) have become important tools in the cardiology arsenal and have shown benefits in several cardiometabolic conditions, through cardioprotective effects and weight loss in addition to diabetes control. As a surrogate of the latter, EAT has been shown to respond favorably to pharmacological modulation by GLP-1 RAs and SGLT2i [37]. A meta-analysis showed that both classes of these medications proved effective in reducing EAT volumes at 3 months and 6 months with a more significant effect seen among younger patients with higher BMI [38]. GLP-1 receptor agonists reduce EAT, likely through direct and indirect metabolic and anti-inflammatory mechanisms. Mechanistically, GLP-1 RAs exert their effects on EAT through several pathways: reducing local adipogenesis, promoting fatty acid oxidation, and potentially inducing white-to-brown adipocyte differentiation within EAT, leading to improved adipocyte metabolism and reduced local inflammation [38, 39]. GLP-1 receptors expressed in human EAT modulate the expression of genes involved in lipid metabolism and inflammation upon activation by GLP-1 RAs, further supporting a direct effect on this fat depot [40].

GLP-1 RA are associated with a reduction in EAT in patients with type 2 diabetes and obesity. Multiple clinical studies and meta-analyses demonstrate that GLP-1 RAs significantly decrease EAT thickness and volume independently of their effects on overall body weight and glycemic control [41, 42]. The reduction in EAT with GLP-1 RA therapy is believed to contribute to their observed cardiovascular benefits, as evidenced by large cardiovascular outcome trials [43, 44]. The magnitude of EAT reduction varies by specific agent and patient population, but the effect remains consistent across studies and is observed with both short- and longer-term GLP-1 RA use [41, 42].

SGLT2i also reduce EAT in patients with type 2 diabetes and obesity. Observations include significant reductions in EAT thickness and volume compared to placebo and conventional hypoglycemic therapies, independent of overall body weight loss. This effect is observed as early as four weeks into treatment and sustained for at least six months [45]. The reduction in EAT by SGLT2i is linked to improvements in glycemic control and decreases in inflammatory markers, including TNF-α. Mechanistically, these agents modulate adipose tissue metabolism, reduce local inflammation, and may enhance favorable adipokine signaling and adipocyte phenotype changes [46–48] Dapagliflozin, frequently studied at a dosage of 10 mg daily, shows consistent and substantial reductions in EAT thickness and volume [49–51]. The reduction in EAT with SGLT2i may contribute to their cardioprotective effects, particularly regarding reduced heart failure events and improved cardiac function [38, 52].

### Surgical Approaches

Metabolic and bariatric surgery has been consistently associated with significant reductions in EAT volume. A systematic review and meta-analysis by Launbo et al. demonstrated that bariatric surgery is one of the most effective interventions for decreasing cardiac fat depots, including EAT, with observed reductions being substantial across various studies [32]. More recently, a systematic review and meta-analysis by Pereira et al. reinforced these findings, showing that 95% of included studies reported significant decreases in EAT following metabolic and bariatric procedures, such as Roux-en-Y gastric bypass and sleeve gastrectomy [53]. Reductions in EAT are thought to contribute to the cardiometabolic benefits observed after bariatric surgery, potentially through decreased local inflammation and reduced paracrine signaling affecting the myocardium and coronary arteries. Collectively, these findings support bariatric surgery not only as a weight-loss intervention but also as an effective strategy for reducing cardiac ectopic fat burden, which may partially mediate its cardiovascular benefits.

## Conclusion

In conclusion, “pericardial fat” – comprised of epicardial, pericardial, and paracardial depots – plays a unique role in cardiometabolic health and disease. Advances in imaging and metabolomics have enhanced our ability to quantify these depots and understand their contributions to cardiometabolic pathways. EAT has emerged as a modifiable risk factor associated with CAD, HFpEF, AF, and ventricular arrhythmias. A growing body of evidence demonstrates that lifestyle modification, pharmacologic therapies, and metabolic and bariatric surgery can significantly reduce EAT burden, potentially improving cardiac outcomes. Although observational and interventional studies support these associations, randomized trials are still needed to determine whether reducing EAT directly improves clinical outcomes, or if its reduction simply reflects broader improvements in cardiometabolic health. Until such evidence is available, EAT should be considered as a factor in the context of the patient’s overall risk for cardiometabolic conditions. In the future, managing EAT may be incorporated into broader cardiometabolic risk reduction strategies, emphasizing weight management, metabolic optimization, and lifestyle to reduce the burden of visceral obesity and ectopic fat on cardiovascular health.

## Key References

- Iacobellis G. Epicardial adipose tissue in contemporary cardiology. Nature Reviews Cardiology. 2022;19:593–606.
  - This review provides an in-depth discussion of epicardial adipose tissue biology and relation to cardiovascular diseases.
- Neeland IJ, Fang Zhu, Goncalo Graca, Lymperopoulos A, Iacobellis G, Farzaneh A, et al. Metabolomics Profiling of Epicardial Adipose Tissue: MESA and the Rotterdam Study. J Am Heart Assoc. 2025;14:39750.
  - Findings from this study suggest that epicardial adipose tissue is associated with a distinct metabolomic signature.

## Data Availability

Not applicable. There are no data generated in this manuscript. It is a review article.

## References

[1] Benkovic M, Galić I, Harijan M, Pižurica A. Recent progress in epicardial and pericardial adipose tissue segmentation and quantification based on deep learning: A systematic review. Appl Sci. 2022;12:5217.

[2] Talman AH, Psaltis PJ, Cameron JD, Meredith IT, Seneviratne SK, Wong DTL. Epicardial adipose tissue: far more than a fat depot. Cardiovasc Diagn Ther. 2014;4:416. 25610800 10.3978/j.issn.2223-3652.2014.11.05 PMC4278038

[3] Iacobellis G, Corradi D, Sharma AM. Epicardial adipose tissue: Anatomic, biomolecular and clinical relationships with the heart. Nat Clin Pract Cardiovasc Med [Internet]. 2005 [cited 2025 Jul 2];2:536–43. Available from: https://pubmed.ncbi.nlm.nih.gov/16186852/10.1038/ncpcardio0319 16186852

[4] Iacobellis G. Epicardial adipose tissue in contemporary cardiology. Nature Reviews Cardiology 2022 19:9 [Internet]. 2022 [cited 2025 Jun 30];19:593–606. Available from: https://www.nature.com/articles/s41569-022-00679-9 10.1038/s41569-022-00679-9 PMC8926097 35296869

[5] Napoli G, Pergola V, Basile P, De Feo D, Bertrandino F, Baggiano A, et al. Epicardial and Pericoronary Adipose Tissue, Coronary Inflammation, and Acute Coronary Syndromes. Journal of Clinical Medicine 2023, Vol 12, Page 7212 [Internet]. 2023 [cited 2025 Jul 3];12:7212. Available from: https://www.mdpi.com/2077-0383/12/23/7212/htm 10.3390/jcm12237212 PMC10707039 38068263

[6] Song Y, Tan Y, Deng M, Shan W, Zheng W, Zhang B, et al. Epicardial adipose tissue, metabolic disorders, and cardiovascular diseases: recent advances classified by research methodologies. MedComm (Beijing) [Internet]. 2023 [cited 2025 Jul 3];4:e413. Available from: https://pmc.ncbi.nlm.nih.gov/articles/PMC10594046/10.1002/mco2.413 PMC10594046 37881786

[7] Iacobellis G, Barbaro G. Epicardial adipose tissue feeding and overfeeding the heart. Nutrition [Internet]. 2019 [cited 2025 Jul 3];59:1–6. Available from: https://www.sciencedirect.com/science/article/abs/pii/S0899900718304349?utm_source=chatgpt.com 10.1016/j.nut.2018.07.002 30415157

[8] Willar B, Tran K, Van, Fitzgibbons TP. Epicardial adipocytes in the pathogenesis of atrial fibrillation: an update on basic and translational studies. Front Endocrinol (Lausanne). 2023;14:1154824. 37020587 10.3389/fendo.2023.1154824 PMC10067711

[9] Nalliah CJ, Bell JR, Raaijmakers AJA, Waddell HM, Wells SP, Bernasochi GB, et al. Epicardial Adipose Tissue Accumulation Confers Atrial Conduction Abnormality. J Am Coll Cardiol [Internet]. 2020 [cited 2025 Jul 3];76:1197–211. Available from: https://pubmed.ncbi.nlm.nih.gov/32883413/10.1016/j.jacc.2020.07.017 32883413

[10] Petrosino JM, Longenecker JZ, Ramkumar S, Xu X, Dorn LE, Bratasz A et al. Paracardial fat remodeling affects systemic metabolism through alcohol dehydrogenase 1. J Clin Invest [Internet]. 2021 [cited 2025 Aug 20];131. Available from: 10.1172/JCI145969 PMC7880313 33586683

[11] El Khoudary SR, Shields KJ, Janssen I, Budoff MJ, Everson-Rose SA, Powell LH, et al. Postmenopausal women with greater paracardial fat have more coronary artery calcification than premenopausal women: The Study of Women’s Health Across the Nation (SWAN) cardiovascular fat ancillary study. J Am Heart Assoc [Internet]. 2017 [cited 2025 Aug 20];6. Available from: /doi/pdf/10.1161/JAHA.116.004545?download = true 10.1161/JAHA.116.004545 PMC5523758 28137715

[12] Scherer M, Montoliu I, Qanadli SD, Collino S, Rezzi S, Kussmann M, et al. Blood plasma lipidomic signature of epicardial fat in healthy obese women. Obesity. 2015;23:130–7. 10.1002/oby.20925. 25400283

[13] Neeland IJ, Zhu; F, Lymperopoulos GG, Iacobellis A, Farzaneh G, et al. Metabolomics Profiling of Epicardial Adipose Tissue: MESA and the Rotterdam Study. J Am Heart Assoc [Internet]. 2025;14:39750. 10.1161/JAHA.124.039750?download=true . PMC12449973 40530511

[14] Neeland IJ, Boone SC, Mook-Kanamori DO, Ayers C, Smit RAJ, Tzoulaki I et al. Metabolomics Profiling of Visceral Adipose Tissue: Results From MESA and the NEO Study. J Am Heart Assoc [Internet]. 2019 [cited 2025 Jul 3];8. Available from: 10.1161/JAHA.118.010810 PMC6512086 31017036

[15] Poirier P, Lavie CJ, Connelly K. Practical Implications of Obesity for Cardiovascular Diagnostics. Can J Cardiol. 2025 Jul 12:S0828-282X(25)00540-9. 10.1016/j.cjca.2025.07.006. Epub ahead of print. PMID: 40659314. 40659314

[16] Iacobellis G, Assael F, Ribaudo MC, Zappaterreno A, Alessi G, Di Mario U, et al. Epicardial fat from echocardiography: a new method for visceral adipose tissue prediction. Obes Res. 2003;11(2):304–10. 10.1038/oby.2003.45. 12582228

[17] Parisi V, Petraglia L, Formisano R, Caruso A, Grimaldi MG, Bruzzese D, et al. Validation of the echocardiographic assessment of epicardial adipose tissue thickness at the Rindfleisch fold for the prediction of coronary artery disease. Nutrition, Metabolism and Cardiovascular Diseases [Internet]. 2020;30:99–105. 31648886 10.1016/j.numecd.2019.08.007

[18] Saura D, Oliva MJ, Rodríguez D, Pascual-Figal DA, Hurtado JA, Pinar E, et al. Reproducibility of echocardiographic measurements of epicardial fat thickness. Int J Cardiol [Internet]. 2010;141:311–3. 19110328 10.1016/j.ijcard.2008.11.127

[19] Iacobellis G, Willens HJ. Echocardiographic Epicardial Fat: A Review of Research and Clinical Applications. Journal of the American Society of Echocardiography [Internet]. 2025;22:1311–9. 10.1016/j.echo.2009.10.013 19944955

[20] Benčević M, Galić I, Habijan M, Pižurica A. Recent Progress in Epicardial and Pericardial Adipose Tissue Segmentation and Quantification Based on Deep Learning: A Systematic Review. Applied Sciences 2022. 2022;12:5217.

[21] Ding J, Hsu FC, Harris TB, Liu Y, Kritchevsky SB, Szklo M, et al. The Multi-Ethnic Study of Atherosclerosis (MESA). American Journal of Clinical Nutrition [Internet]. 2009;90:499–504. 19571212 10.3945/ajcn.2008.27358 PMC2728641

[22] Grutta L La, Toia P, Farruggia A, Albano D, Grassedonio E, Palmeri A, et al. Quantification of epicardial adipose tissue in coronary calcium score and CT coronary angiography image data sets: comparison of attenuation values, thickness and volumes. Br J Radiol [Internet]. 2016 [cited 2025 Jul 4];89:20150773. Available from: https://pmc.ncbi.nlm.nih.gov/articles/PMC5258148/10.1259/bjr.20150773 PMC5258148 26987374

[23] Guglielmo M, Penso M, Carerj ML, Giacari CM, Volpe A, Fusini L, et al. DEep LearnIng-based QuaNtification of epicardial adipose tissue predicts MACE in patients undergoing stress CMR. Atherosclerosis [Internet]. 2024 [cited 2025 Jul 4];397:117549. Available from: https://www.sciencedirect.com/science/article/pii/S0021915024001096?casa_token=LM7WuKJY07gAAAAA:EbaNaMJZI7sfS22on_82RdDOpwB1L_D3tjS160FkK4vhyjsEEOJ_C-NTETw9X3UZeeAK7iYHOg 10.1016/j.atherosclerosis.2024.117549 38679562

[24] Chong B, Jayabaskaran J, Ruban J, Goh R, Chin YH, Kong G, et al. Epicardial Adipose Tissue Assessed by Computed Tomography and Echocardiography Are Associated with Adverse Cardiovascular Outcomes: A Systematic Review and Meta-Analysis. Circ Cardiovasc Imaging [Internet]. 2023 [cited 2025 Jul 5];16:E015159. Available from: https://www.ahajournals.org/doi/pdf/10.1161/CIRCIMAGING.122.015159?download=true 10.1161/CIRCIMAGING.122.015159 37192298

[25] Eisenberg E, McElhinney PA, Commandeur F, Chen X, Cadet S, Goeller M, et al. Deep Learning-Based Quantification of Epicardial Adipose Tissue Volume and Attenuation Predicts Major Adverse Cardiovascular Events in Asymptomatic Subjects. Circ Cardiovasc Imaging [Internet]. 2020;13:E009829. 10.1161/CIRCIMAGING.119.009829?download=true . 32063057 PMC8422968

[26] Wu A, Yang Z, Zhang X, Lin Z, Lu H. Association Between Epicardial Adipose Tissue and Left Atrial and Ventricular Function in Patients With Heart Failure: A Systematic Review and Meta-Analysis. Curr Probl Cardiol [Internet]. 2023;48:101979. 37481217 10.1016/j.cpcardiol.2023.101979

[27] Chang T-Y, Veterans General Hospital T, Cristina S, Leandro AC, Lu Liu C, Zhang S, Wu Q et al. Prognostic value of epicardial adipose tissue in heart failure: a systematic review and meta-analysis. Front Cardiovasc Med [Internet]. 2025 [cited 2025 Jul 5];12:1618614. Available from: https://www.frontiersin.org/articles/10.3389/fcvm.2025.1618614 PMC12240945 40642751

[28] Chen J, Mei Z, Yang Y, Dai C, Wang Y, Zeng R, et al. Epicardial adipose tissue is associated with higher recurrence risk after catheter ablation in atrial fibrillation patients: a systematic review and meta-analysis. BMC Cardiovasc Disord. 2022;22:1–10. 10.1186/s12872-022-02703-9. 35690712 PMC9188706

[29] Lavie CJ, Hegele RA, Poirier P, Thompson JA. Obesity in Cardiovascular Disease Prevention and Treatment—A Topic Getting Heavier as We Speak. Can J Cardiol. 2025 Jul 15:S0828-282X(25)00545-8. 10.1016/j.cjca.2025.07.011. Epub ahead of print. PMID: 40675447. 40675447

[30] Sepehri Shamloo A, Schoene K, Stauber A, Darma A, Dagres N, Dinov B et al. Epicardial adipose tissue thickness as an independent predictor of ventricular tachycardia recurrence following ablation. Heart Rhythm [Internet]. 2019 [cited 2025 Jul 5];16:1492–8. Available from: https://pubmed.ncbi.nlm.nih.gov/31202898/10.1016/j.hrthm.2019.06.009 31202898

[31] Iacobellis G, Singh N, Wharton S, Sharma AM. Substantial changes in epicardial fat thickness after weight loss in severely obese subjects. Obesity [Internet]. 2008 [cited 2025 Jul 5];16:1693–7. Available from: https://pubmed.ncbi.nlm.nih.gov/18451775/10.1038/oby.2008.251 18451775

[32] Launbo N, Zobel EH, von Scholten BJ, Færch K, Jørgensen PG, Christensen RH. Targeting epicardial adipose tissue with exercise, diet, bariatric surgery or pharmaceutical interventions: A systematic review and meta-analysis. Obesity Reviews [Internet]. 2021 [cited 2025 Jul 5];22. Available from: https://pubmed.ncbi.nlm.nih.gov/32896056/10.1111/obr.13136 32896056

[33] Colonetti T, Grande AJ, Amaral MC, Colonetti L, Uggioni ML, da Rosa MI et al. Effect of exercise on epicardial adipose tissue in adults: a systematic review and meta-analyses. Heart Fail Rev [Internet]. 2021 [cited 2025 Jul 5];26:1399–411. Available from: https://link.springer.com/article/10.1007/s10741-020-09965-5 32418010

[34] Piercy KL, Troiano RP, Ballard RM, Carlson SA, Fulton JE, Galuska DA et al. The physical activity guidelines for Americans. JAMA - Journal of the American Medical Association [Internet]. 2018 [cited 2025 Jul 5];320:2020–8. Available from: https://pubmed.ncbi.nlm.nih.gov/30418471/10.1001/jama.2018.14854 PMC9582631 30418471

[35] Rivas Galvez RE, Morales Portano JD, Trujillo Cortes R, Gomez Alvarez EB, Sanchez Cubias SM, Zelaya SM. Reduction of epicardial adipose tissue thickness with PCSK9 inhibitors. Eur Heart J. 2020. 10.1093/ehjci/ehaa946.3008.

[36] Sardà H, Colom C, Benitez S, Carreras G, Amigó J, Miñambres I et al. PCSK9 plasma concentration is associated with epicardial adipose tissue volume and metabolic control in patients with type 1 diabetes. Sci Rep [Internet]. 2024 [cited 2025 Jul 5];14. Available from: https://pubmed.ncbi.nlm.nih.gov/38532033/10.1038/s41598-024-57708-5 PMC10965929 38532033

[37] Iacobellis G, Baroni MG. Cardiovascular risk reduction throughout GLP-1 receptor agonist and SGLT2 inhibitor modulation of epicardial fat. J Endocrinol Invest [Internet]. 2022;45:489–95. 34643917 10.1007/s40618-021-01687-1

[38] Myasoedova VA, Parisi V, Moschetta D, Valerio V, Conte M, Massaiu I et al. Efficacy of cardiometabolic drugs in reduction of epicardial adipose tissue: a systematic review and meta-analysis. Cardiovasc Diabetol [Internet]. 2023 [cited 2025 Jul 5];22:1–11. Available from: https://cardiab.biomedcentral.com/articles/ https://doi.org/10.1186/s12933-023-01738-2 10.1186/s12933-023-01738-2 PMC9890718 36721184

[39] Dozio E, Vianello E, Malavazos AE, Tacchini L, Schmitz G, Iacobellis G, et al. Epicardial adipose tissue GLP-1 receptor is associated with genes involved in fatty acid oxidation and white-to-brown fat differentiation: A target to modulate cardiovascular risk? Int J Cardiol [Internet]. 2019;292:218–24. 31023563 10.1016/j.ijcard.2019.04.039

[40] Pop ASK, Dănilă MD, Giuchici S, Buriman DG, Lolescu BM, Sturza A, et al. Epicardial adipose tissue as target of the incretin-based therapies in cardio-metabolic pathologies: a narrative review. Can J Physiol Pharmacol [Internet]. 2025;103:182–92. 40048723 10.1139/cjpp-2024-0384

[41] Berg G, Barchuk M, Lobo M, Nogueira JP. Effect of glucagon-like peptide-1 (GLP-1) analogues on epicardial adipose tissue: A meta-analysis. Diabetes and Metabolic Syndrome: Clinical Research and Reviews [Internet]. 2022 [cited 2025 Aug 20];16. Available from: https://pubmed.ncbi.nlm.nih.gov/35816950/ 10.1016/j.dsx.2022.102562 35816950

[42] Morano S, Romagnoli E, Filardi T, Nieddu L, Mandosi E, Fallarino M, et al. Short-term effects of glucagon-like peptide 1 (GLP-1) receptor agonists on fat distribution in patients with type 2 diabetes mellitus: an ultrasonography study. Acta Diabetol [Internet]. 2015;52:727–32. 25577244 10.1007/s00592-014-0710-z

[43] Nauck MA, Meier JJ, Cavender MA, El Aziz MA, Drucker DJ. Cardiovascular actions and clinical outcomes with glucagon-like peptide-1 receptor agonists and dipeptidyl peptidase-4 inhibitors. Circulation [Internet]. 2017;136:849–70. 10.1161/CIRCULATIONAHA.117.028136?download=true . 28847797

[44] Ussher JR, Drucker DJ. Glucagon-like peptide 1 receptor agonists: cardiovascular benefits and mechanisms of action. Nat Rev Cardiol [Internet]. 2023;20:463–74. 36977782 10.1038/s41569-023-00849-3

[45] Bao Y, Hu Y, Shi M, Zhao Z. SGLT2 inhibitors reduce epicardial adipose tissue more than GLP-1 agonists or exercise interventions in patients with type 2 diabetes mellitus and/or obesity: a systematic review and network meta-analysis. Diabetes Obes Metab. 2025;27:1096–112. 10.1111/dom.16107. 39639835

[46] Morciano C, Gugliandolo S, Capece U, Di Giuseppe G, Mezza T, Ciccarelli G et al. SGLT2 inhibition and adipose tissue metabolism: current outlook and perspectives. Cardiovascular Diabetology 2024 23:1 [Internet]. 2024 [cited 2025 Aug 20];23:1–12. Available from: https://cardiab.biomedcentral.com/articles/ https://doi.org/10.1186/s12933-024-02539-x 10.1186/s12933-024-02539-x PMC11660748 39702365

[47] Pandey AK, Bhatt DL, Pandey A, Marx N, Cosentino F, Pandey A et al. Mechanisms of benefits of sodium-glucose cotransporter 2 inhibitors in heart failure with preserved ejection fraction. Eur Heart J [Internet]. 2023 [cited 2025 Aug 20];44:3640–51. Available from: https://pubmed.ncbi.nlm.nih.gov/37674356/ 10.1093/eurheartj/ehad389 37674356

[48] Bakkar NMZ, AlZaim I, El-Yazbi AF. Depot-specific adipose tissue modulation by SGLT2 inhibitors and GLP1 agonists mediates their cardioprotective effects in metabolic disease. Clin Sci. 2022;136:1631–51. 10.1042/CS20220404 36383188

[49] Cinti F, Leccisotti L, Sorice GP, Capece U, D’Amario D, Lorusso M, et al. Dapagliflozin treatment is associated with a reduction of epicardial adipose tissue thickness and epicardial glucose uptake in human type 2 diabetes. Cardiovasc Diabetol. 2023;22:1–10. 38115004 10.1186/s12933-023-02091-0 PMC10731727

[50] Sato T, Aizawa Y, Yuasa S, Kishi S, Fuse K, Fujita S et al. The effect of dapagliflozin treatment on epicardial adipose tissue volume. Cardiovasc Diabetol [Internet]. 2018 [cited 2025 Aug 20];17:1–9. Available from: https://cardiab.biomedcentral.com/articles/ 10.1186/s12933-017-0658-8 PMC5753537 29301516

[51] Song X, ting, Wei Ylong, Rui Y, fei, Fan L. Echocardiographic evaluation of the effect of dapagliflozin on epicardial adipose tissue and left ventricular systolic function in type 2 diabetes mellitus. J Diabetes Complications [Internet]. 2023 [cited 2025 Aug 20];37. Available from: https://pubmed.ncbi.nlm.nih.gov/37235925/ 10.1016/j.jdiacomp.2023.108509 37235925

[52] Braunwald E. Gliflozins in the management of cardiovascular disease. N Engl J Med. 2022;386:2024–34. 10.1056/NEJMra2115011. 35613023

[53] Pereira JPS, Calafatti M, Martinino A, Ramnarain D, Stier C, Parmar C, et al. Epicardial adipose tissue changes after bariatric and metabolic surgery: a systematic review and meta-analysis. Obes Surg. 2023;33:3636–48. 10.1007/s11695-023-06848-0. 37801237

